# Tubular HIPK2 is a key contributor to renal fibrosis

**DOI:** 10.1172/jci.insight.136004

**Published:** 2020-09-03

**Authors:** Wenzhen Xiao, Jing E, Li Bao, Ying Fan, Yuanmeng Jin, Andrew Wang, David Bauman, Zhengzhe Li, Ya-Li Zheng, Ruijie Liu, Kyung Lee, John Cijiang He

**Affiliations:** 1 Division of Nephrology, Department of Medicine, Icahn School of Medicine at Mount Sinai, New York, New York, USA.; 2 Department of Nephrology, People’s Hospital of Ningxia Hui Autonomous Region, Ningxia, China.; 3 Department of Nephrology, Shanghai Jiao Tong University Affiliated Sixth People’s Hospital, Shanghai, China.; 4 Department of Nephrology, Ruijin Hospital, Shanghai Jiao Tong University, Shanghai, China.

**Keywords:** Nephrology, Chronic kidney disease, Fibrosis

## Abstract

We previously used global *Hipk2*-null mice in various models of kidney disease to demonstrate the central role of homeodomain-interacting protein kinase 2 (HIPK2) in renal fibrosis development. However, renal tubular epithelial cell–specific (RTEC-specific) HIPK2 function in renal fibrogenesis has yet to be determined. Here, we show that modulation of tubular HIPK2 expression and activity affects renal fibrosis development in vivo. The loss of HIPK2 expression in RTECs resulted in a marked diminution of renal fibrosis in unilateral ureteral obstruction (UUO) mouse models and HIV-associated nephropathy (HIVAN) mouse models, which was associated with the reduction of Smad3 activation and downstream expression of profibrotic markers. Conversely, WT HIPK2 overexpression in RTECs accentuated the extent of renal fibrosis in the setting of UUO, HIVAN, and folic acid–induced nephropathy in mice. Notably, kinase-dead HIPK2 mutant overexpression or administration of BT173, an allosteric inhibitor of HIPK2-Smad3 interaction, markedly attenuated the renal fibrosis in these mouse models of kidney disease, indicating that HIPK2 requires both the kinase activity and its interaction with Smad3 to promote TGF-β–mediated renal fibrosis. Together, these results establish an important RTEC-specific role of HIPK2 in kidney fibrosis and further substantiate the inhibition of HIPK2 as a therapeutic approach against renal fibrosis.

## Abstract 2

Modulation of HIPK2 expression in murine renal tubular epithelial cells reveals an important role in renal fibrosis development.

## Introduction

Renal fibrosis is the final convergent pathway for progressive chronic kidney diseases (CKDs) regardless of the original etiologies of the disease (1). The extent of renal fibrosis is not only a marker of kidney injury but also predicts the loss of function and progression of damage in the kidney (2–4). Renal fibrogenesis involves cells from all compartments of the kidney. Although ample information exists on the molecular mechanisms underlying renal fibrogenesis, there is still a paucity of success in translating this knowledge to clinical application (5, 6). Therefore, better understanding of these mechanisms and development of potential drugs for kidney fibrosis are urgently needed.

Recent evidence suggests that TGF-β, Wnt, and Notch signaling pathways promote fibrosis in the setting of kidney disease (7–10). However, their inhibitors have not yet been tested or proven successful in clinical trials (11). Using a combination of computational and experimental approaches, we previously identified homeodomain-interacting protein kinase 2 (HIPK2) as an essential mediator of kidney fibrosis (12). HIPK2 expression was elevated in various human kidney diseases and associated with renal fibrosis progression (12). We showed that it promotes renal fibrosis by potentiating the signaling cascades of TGF-β, Wnt, Notch, and NF-κB pathways. The central role of HIPK2 was previously confirmed from observations that the genetic ablation of *Hipk2* in mice (12) and BT173, a small molecule inhibitor of HIPK2 (13), markedly attenuated kidney fibrosis and improved kidney function in experimental models of kidney disease. Although these data strongly support the role of HIPK2 as a key driver of fibrosis in kidney disease, the effect of HIPK2 function in renal tubular epithelial cells (RTECs) or other cell types in this process was unclear. As BT173 blocked the function of HIPK2 by an allosteric mechanism altering the protein-protein interaction between HIPK2 and Smad3, without interfering with its kinase function, it was also unclear whether the kinase activity was required for its profibrotic effects in vivo. To address these questions, we generated 3 lines of tetracycline-inducible RTEC-specific HIPK2 transgenic mice: inducible RTEC-specific HIPK2-KO, HIPK2 WT overexpression, and HIPK2 kinase-dead mutant mice. Our results indicate that the RTEC function of HIPK2 is a key mediator of renal fibrosis, and that increased HIPK2 expression and kinase activity are required for kidney fibrosis progression.

## Results

### RTEC-specific KO of HIPK2 mitigates renal fibrosis development.

To assess the RTEC-specific role of HIPK2 in vivo, we generated inducible RTEC-specific *Hipk2*-KO mice by crossing *Hipk2^fl/fl^* mice with transgenic mice expressing Pax8-driven reverse tetracycline transactivator (Pax8-rtTA) and Cre recombinase under the control of tetracycline-responsive element (TRE-Cre), resulting in *Hipk2^fl/fl^* Pax8-rtTA TRE-Cre mice. The effective ablation of *Hipk2* in RTECs in *Hipk2^fl/fl^* Pax8-rtTA TRE-Cre mice fed with doxycycline-supplemented (dox-supplemented) chow for 3 weeks (Pax8-HIPK2^–/–^ mice) was confirmed by Western blot of kidney cortices and compared with those of control *Hipk2^fl/fl^* Pax8-rtTA TRE-Cre mice fed with normal chow without dox (Pax8-HIPK2^+/+^ mice) (Supplemental Figure 1; supplemental material available online with this article; https://doi.org/10.1172/jci.insight.136004DS1).

**Figure 1:**
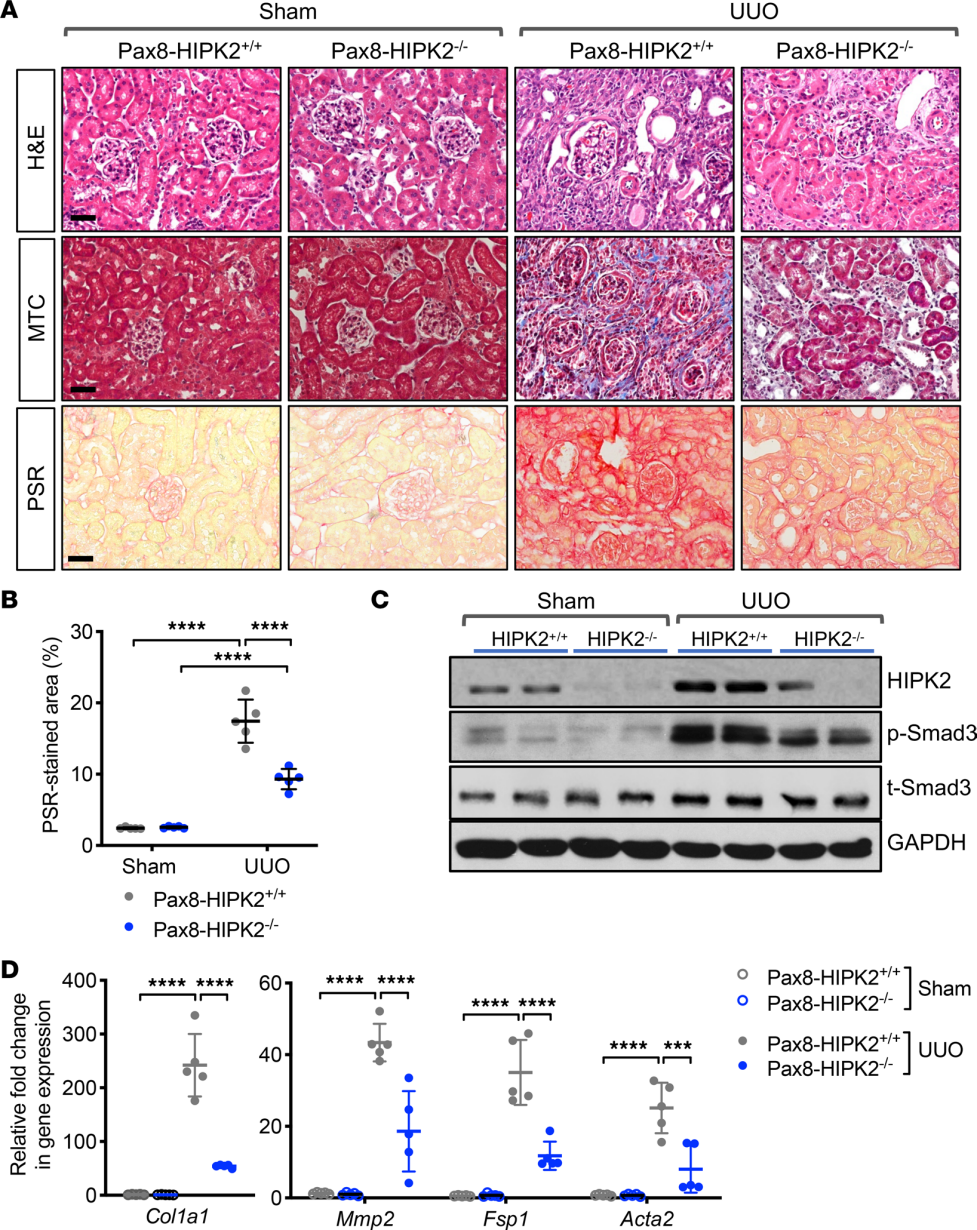
RTEC-specific ablation of HIPK2 attenuates fibrosis in UUO kidneys. **Six-week-old***Hipk2*^fl/fl^ Pax8-rtTA TRE-Cre mice were fed with control chow (Pax8-HIPK2^+/+^) or doxycycline-supplemented chow (Pax8-HIPK2^–/–^) for 3 weeks before UUO surgery (*n* = 5 in each group). Kidneys from UUO or sham surgery were analyzed 14 days after surgery. (**A**) Representative images of H&E-, Masson’s trichrome (MCT)-, or picrosirius red (PSR)-stained kidneys are shown. Scale bars: 50 μm. (**B**) Average percentage of PSR-stained area per mouse (*n* = 5 mice per group). (**C**) Representative Western blot analysis of Pax8-HIPK2^+/+^ and Pax8-HIPK2^–/–^ mouse kidney cortices for the expression of HIPK2, phospho-Smad3 (p-Smad3), and total Smad3 (t-Smad3) proteins. (**D**) Real-time qPCR analysis of fibrosis markers in kidney cortices. Data are presented as mean ± SD. ***P < 0.001 and ****P < 0.0001 when compared between indicated groups by 1-way ANOVA with Tukey’s multiple comparison test. RTEC, renal tubular epithelial cell; UUO, unilateral ureteral obstruction.

The effect of HIPK2 loss in RTECs was first tested with a unilateral ureteral obstruction (UUO) model of renal fibrosis. Nine-week-old Pax8-HIPK2^–/–^ and control Pax8-HIPK2^+/+^ mice were subjected to sham or UUO surgery (*n* = 5 mice per group). All mice were euthanized 14 days after surgery. There were no notable changes in the body weight of mice between groups by dox feeding or surgery (data not shown). As anticipated, the loss of HIPK2 in RTECs had no effects in sham-operated kidneys; however, it resulted in a marked attenuation of renal fibrosis development following UUO when compared with control Pax8-HIPK2^+/+^ UUO mice (Figure 1, A and B). Western blot of kidney cortices showed that the total level of HIPK2 was elevated in response to UUO kidneys in comparison with the sham control kidneys, consistent with our previous observation (12) (Figure 1C). The activation of Smad3, as assessed by phosphorylation of Smad3 (S423/425), was also markedly increased in UUO kidneys in comparison with sham control kidneys but was reduced in Pax8-HIPK2^–/–^ UUO kidneys in comparison with the Pax8-HIPK2^+/+^ UUO kidneys (Figure 1C). Pax8-HIPK2^–/–^ UUO kidneys also had attenuated expression of profibrotic marker genes in comparison with the control UUO kidneys (Figure 1D).

### RTEC-specific overexpression of HIPK2 aggravates renal fibrosis in UUO and Tg26 mice.

Considering that RTEC-specific loss of HIPK2 attenuated the renal fibrosis, we next explored whether RTEC-specific overexpression of HIPK2 could conversely augment renal fibrosis. Transgenic mice expressing a full-length human HIPK2 under the regulation of a TRE (TRE-hHIPK2 WT, Supplemental Figure 2A) were generated in the FVB/N inbred strain as described in the Methods and crossed with Pax8-rtTA mice (FVB/N) to generate Pax8-rtTA TRE-hHIPK2 WT double transgenic mice. Three weeks of dox feeding led to robust induction of HIPK2 expression (~3-fold increase) in kidney cortices of Pax8-rtTA TRE-hHIPK2 WT mice ( Pax8-HIPK2^WT^ mice) in comparison with Pax8-HIPK2^+/+^ mice (Supplemental Figure 2, C and D).

**Figure 2:**
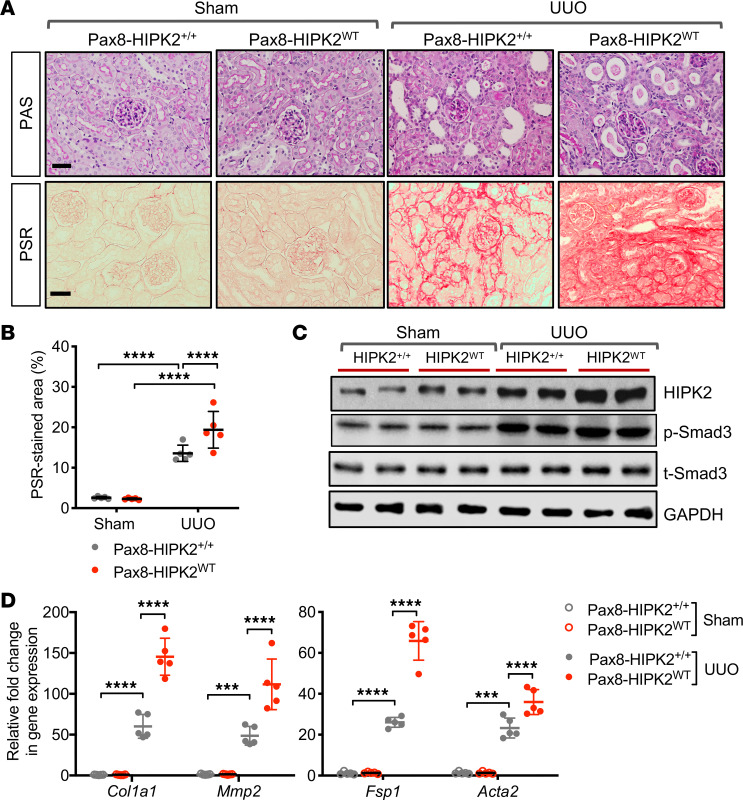
RTEC overexpression of HIPK2 exacerbates fibrosis in UUO kidneys. **Six-week-old** Pax8-rtTA TRE-hHIPK2 WT mice were fed with control chow (Pax8-HIPK2^+/+^) or doxycycline-supplemented chow (Pax8-HIPK2^WT^) for 3 weeks before UUO surgery (*n* = 5 in each group). Kidneys from UUO or sham surgery were analyzed 14 days after surgery. (**A**) Images of periodic acid-Schiff (PAS)- and picrosirius red (PSR)-stained mouse kidneys with sham or UUO surgery. (**B**) Average percentage of PSR-stained area per mouse (*n* = 5 mice per group). (**C**) Representative Western blot images of Pax8-HIPK2^+/+^ and Pax8-HIPK2^WT^ mouse kidney cortices. (**D**) Real-time qPCR analysis of fibrosis markers in kidney cortices. Data are presented as mean ± SD. ***P < 0.001, and ****P < 0.0001 when compared between indicated groups by 1-way ANOVA with Tukey’s multiple comparison test.

We next tested the effect of RTEC-specific HIPK2 overexpression in the UUO model as previously described. Nine-week-old Pax8-HIPK2^WT^ and control Pax8-HIPK2^+/+^ mice were subjected to sham or UUO surgery (*n* = 5 mice per group). All mice were euthanized at 10 days after surgery. Although HIPK2 overexpression in RTECs had no observable effect in the sham-operated mice, it markedly augmented the renal fibrosis development after UUO (Figure 2, A and B). This augmentation in the kidneys of Pax8-HIPK2^WT^ UUO mice was associated with enhanced Smad3 phosphorylation and increased expression of profibrotic marker genes in comparison with Pax8-HIPK2^WT^ UUO mice (Figure 2, C and D).

**Figure 3:**
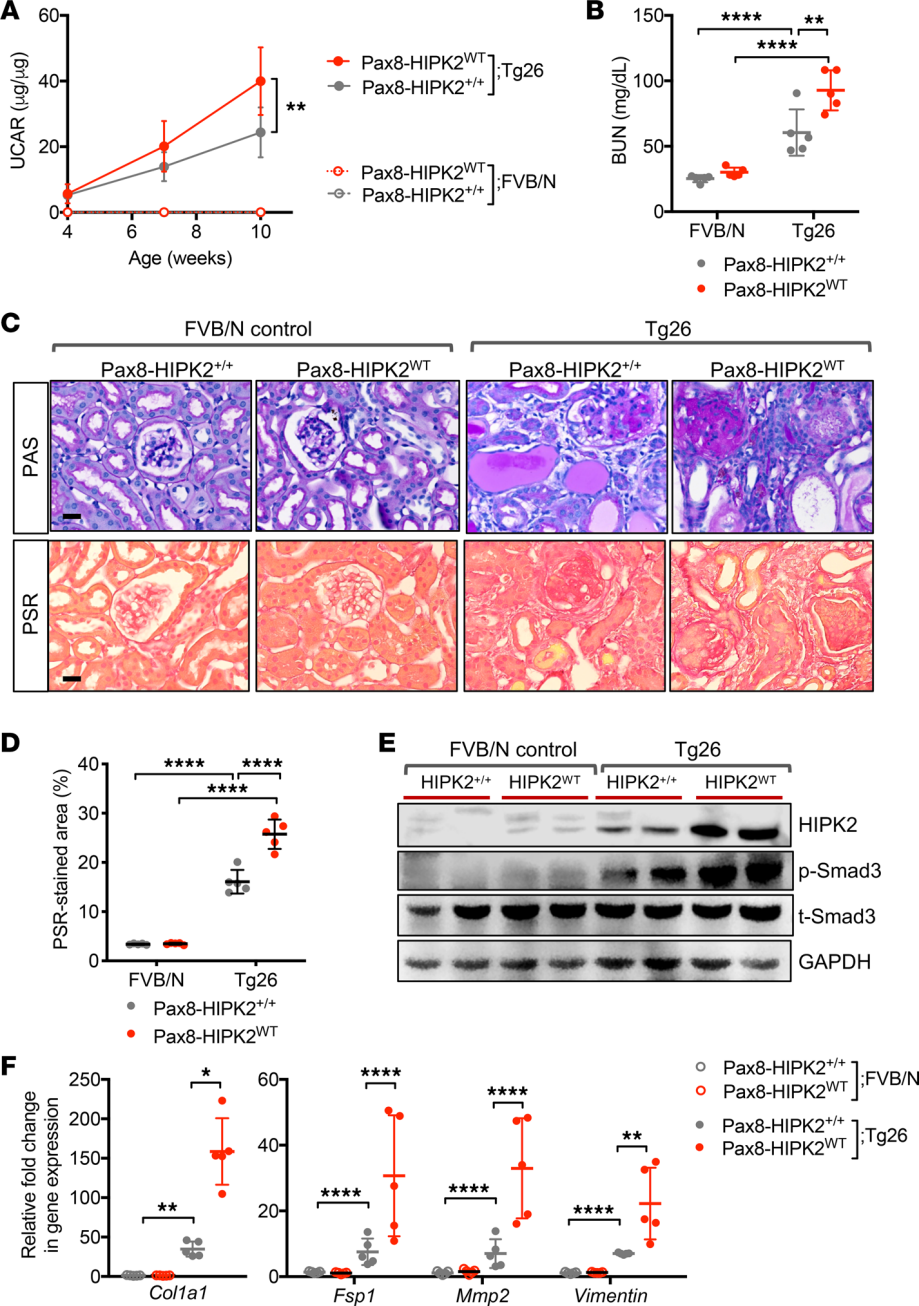
RTEC overexpression of HIPK2 exacerbates renal injury and fibrosis in Tg26 mice. **Four-week-old** Pax8-rtTA TRE-hHIPK2 WT Tg26 mice with established albuminuria were fed with control chow (Pax8-HIPK2^+/+^ Tg26) or doxycycline-supplemented chow (Pax8-HIPK2^WT^ Tg26) for 4 weeks (*n* = 5 in each group). (**A**) Average urinary albumin-to-creatinine ratio (UACR) between mice 4–10 weeks of age in (*n* = 5 mice per group). (**B**) Blood urea nitrogen (BUN) levels in 10-week-old mice. (**C**) Images of PAS- and PSR-stained kidneys. Scale bars: 20 μm. (**D**) Average percentage of PSR-stained area per mouse (*n* = 5 mice per group). (**E**) Representative Western blot images of control and Tg26 mouse cortices. (**F**) qPCR analysis of fibrosis markers. All data are presented as mean ± SD. *P < 0.05, **P < 0.01, and ****P < 0.0001 when compared between indicated groups by 1-way ANOVA with Tukey’s multiple comparison test.

To further validate the RTEC-specific role of HIPK2 in kidney fibrosis, we used a second model of kidney fibrosis, HIV-1 transgenic mice (Tg26 mice). Tg26 mice develop severe glomerulosclerosis and tubulointerstitial fibrosis, mimicking human HIV-associated nephropathy (HIVAN) (14), and serves as an optimal model to study kidney fibrosis in vivo. Pax8-HIPK2^WT^ was crossed with Tg26 mice to generate Pax8-HIPK2^WT^ Tg26 in FVB/N background, and Pax8-HIPK2^WT^ littermates without the HIV-1 transgene were used as controls (FVB/N controls). RTEC HIPK2 expression was induced by dox feeding in 4-week-old mice when albuminuria is already established in Tg26 mice. All mice were euthanized at 10 weeks of age. The induction of HIPK2 expression in RTECs worsened albuminuria and worsened renal function in Pax8-HIPK2^WT^ Tg26 mice in comparison with control Tg26 mice (Figure 3, A and B). Histological analysis showed exacerbated tubulointerstitial injury and fibrosis (Figure 3, C and D). Western blot analysis confirmed that HIPK2 expression was increased in the kidneys of Tg26 mice, and a further increase was observed in the HIPK2 overexpression Tg26 mice (Figure 3E). This was associated with even further enhanced Smad3 phosphorylation and with increased expression of profibrosis markers (Figure 3, E and F). Together with the preceding data on Pax8-HIPK2^–/–^ mice, these results demonstrate that RTEC-specific HIPK2 function is a key determinant in the RTEC injury, ensuing kidney fibrosis, and in renal function in vivo.

### RTEC-specific overexpression of kinase-dead HIPK2 attenuates renal fibrosis.

We next explored whether the kinase activity of HIPK2 was required for its effect on promoting renal fibrosis in vivo by generating a transgenic mouse model with RTEC-specific HIPK2 kinase-dead mutant overexpression. Using the same strategy as previously described, we generated tetracycline-inducible transgenic mice expressing the hHIPK2 kinase-dead mutant (TRE-hHIPK2 KD, Supplemental Figure 2B), which were crossed with Pax8-rtTA transgenic mice to generate Pax8-rtTA TRE-HIPK2 KD mice. As protein overexpression in RTECs could potentially artificially lead to RTEC injury and fibrosis, Pax8-rtTA TRE-HIPK2 KD mice would also serve as an appropriate internal control for the observations made in Pax8-rtTA TRE-HIPK2 WT mice. Western blot analysis confirmed an ~3-fold induction of hHIPK2-KD protein in kidney cortices in Pax8-rtTA TRE-HIPK2 KD mice after 3 weeks of dox feeding (Pax8-HIPK2^WT^ mice) in comparison with those without the dox feeding (Pax8-HIPK2^+/+^ mice) (Supplemental Figure 2, C and D). Therefore, the level of mutant HIPK2 protein induced in Pax8-HIPK2^KD^ kidneys was comparable to that of WT HIPK2 protein in Pax8-HIPK2^WT^ kidneys (Supplemental Figure 2, C and D).

**Figure 4:**
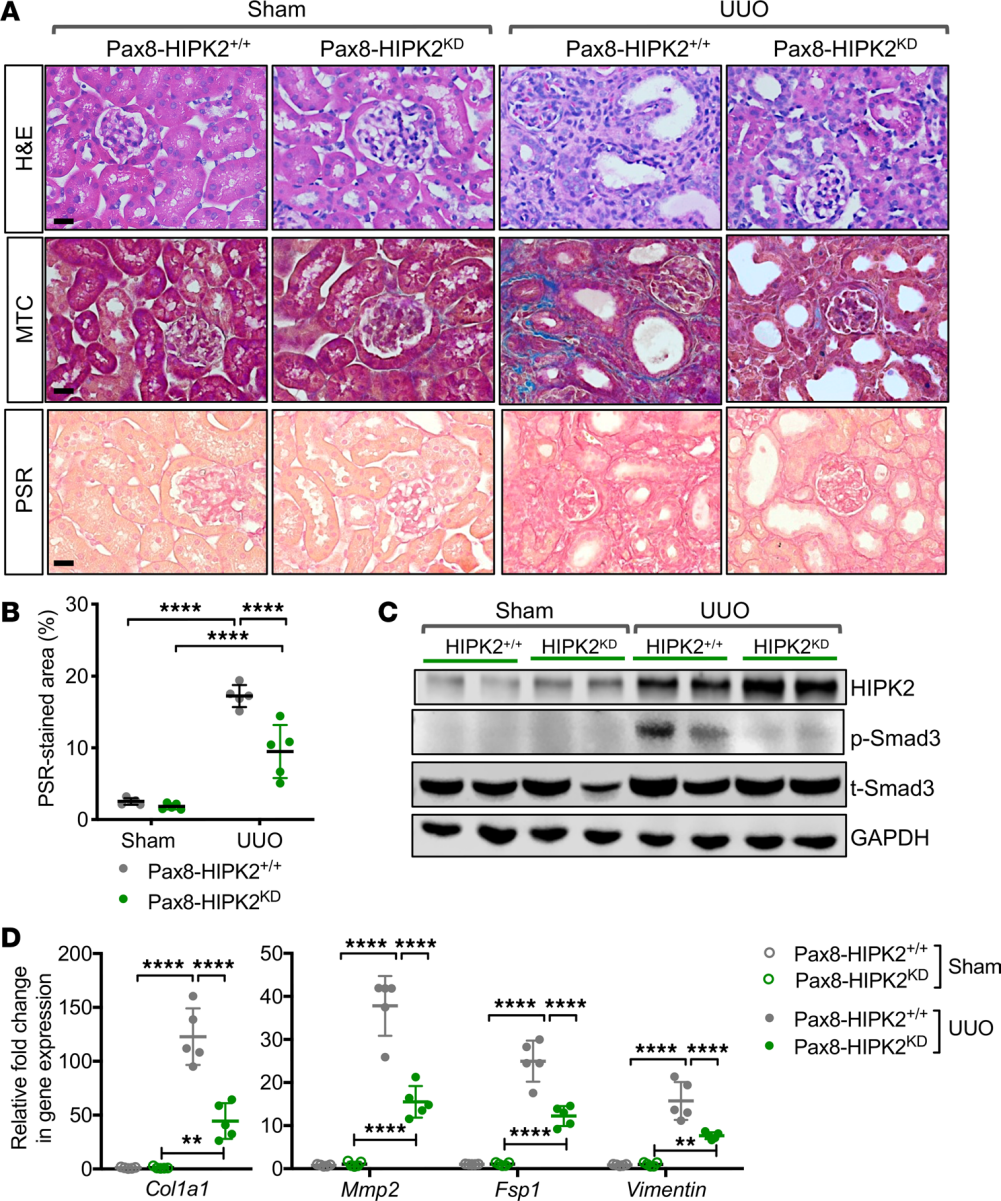
RTEC overexpression of kinase-dead (KD) mutant of HIPK2 attenuates fibrosis in UUO kidneys. **Six-week-old** Pax8-rtTA TRE-hHIPK2 KD mice were fed with control chow (Pax8-HIPK2^+/+^) or doxycycline-supplemented chow (Pax8-HIPK2^KD^) for 3 weeks before UUO surgery (*n* = 5 in each group). Kidneys from UUO or sham surgery were analyzed 14 days after surgery. (**A**) Images of H&E-, MTC-, and PSR-stained mouse kidneys with sham or UUO surgery. Scale bars: 20 μm. (**B**) Percentage of PSR-stained area per field (*n* = 30 randomly selected fields per group). (**C**) Representative Western blot images of Pax8-HIPK2^+/+^ and Pax8-HIPK2^KD^ mouse kidney cortices. (**D**) qPCR analysis of fibrosis markers. Data are presented as mean ± SD. **P < 0.01 and ****P < 0.0001 when compared between indicated groups by 1-way ANOVA with Tukey’s multiple comparison test.

The effect of kinase-dead HIPK2 overexpression in RTECs was then examined in UUO-induced renal fibrosis. After 3 weeks of dox feeding, 9-week-old Pax8-HIPK2^KD^ and control Pax8-HIPK2^+/+^ mice were subjected to sham or UUO surgery (*n* = 5 mice per group). All mice were euthanized at 14 days after surgery. In contrast to the observations in Pax8-HIPK2^WT^ UUO kidneys, the kidneys of Pax8-HIPK2^KD^-UUO mice showed a marked reduction in renal fibrosis (Figure 4, A and B), which was associated with reduced Smad3 activation and expression of profibrosis markers (Figure 4, C and D).

**Figure 5:**
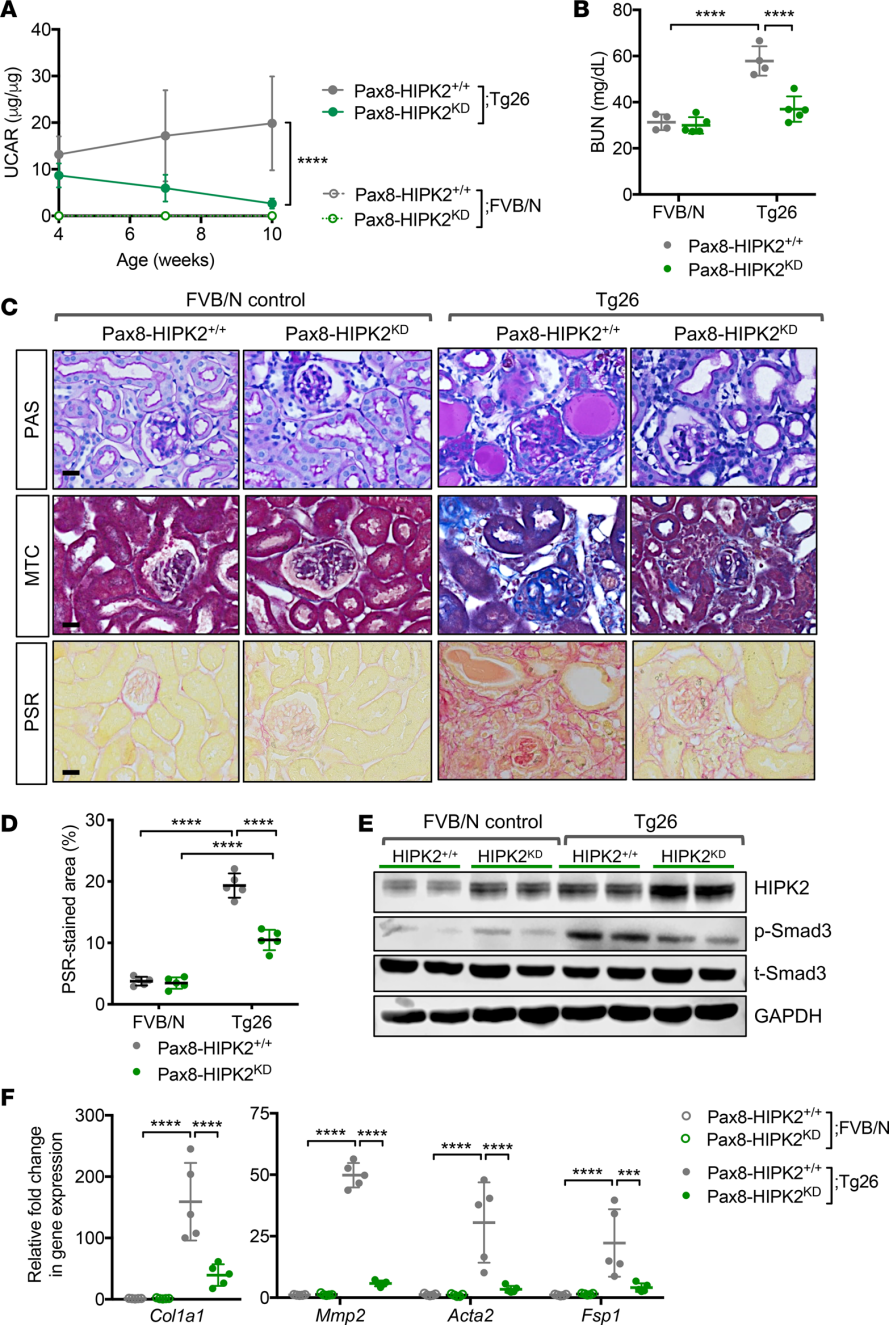
RTEC overexpression of kinase-dead (KD) mutant of HIPK2 attenuates renal injury and fibrosis in Tg26 mice. **Four-week-old** Pax8-rtTA TRE-hHIPK2 KD Tg26 mice with established albuminuria were fed with control chow (Pax8-HIPK2^+/+^ Tg26) or doxycycline-supplemented chow (Pax8-HIPK2^KD^ Tg26) for 4 weeks (*n* = 5 in each group). (**A**) Average urinary albumin-to-creatinine ratio (UACR) for mice between 4 to 10 weeks of age. (**B**) Blood urea nitrogen (BUN) levels in 10-week-old mice. (**C**) Images of PAS- and PSR-stained kidneys. Scale bars: 20 μm. (**D**) Average percentage of PSR-stained area per mouse (*n* = 5 mice per group). (**E**) Representative Western blot images of control and Tg26 mouse cortices. (**F**) qPCR analysis of fibrosis markers. All data are presented as mean ± SD. ***P < 0.001 and ****P < 0.0001 when compared between indicated groups by 1-way ANOVA with Tukey’s multiple comparison test.

We also examined the effect of HIPK2^KD^ tubular overexpression in the setting of HIVAN-induced chronic kidney disease and fibrosis, using Tg26 mice. Although both Pax8-HIPK2^KD^-Tg26 and control Pax8-HIPK2^+/+^ Tg26 mice displayed a similar extent of albuminuria at 4 weeks of age, by 10 weeks of age Pax8-HIPK2^KD^ Tg26 mice had significantly lower urinary albumin (Figure 5A) and BUN levels (Figure 5B) in comparison with Pax8-HIPK2^+/+^ Tg26 mice. The histologic analysis also showed a marked attenuation in tubulointerstitial injury and fibrosis development in Pax8-HIPK2^KD^ Tg26 kidneys in comparison with Pax8-HIPK2^+/+^ Tg26 kidneys (Figure 5, C and D), which were associated with lower levels of Smad3 phosphorylation and profibrosis marker gene expression (Figure 5, E and F). Together, these findings show an essential function of HIPK2 kinase activity in promoting renal fibrosis and suggest that overexpression of HIPK2 kinase-dead mutant protein attenuates renal fibrosis by acting as a dominant-negative against the endogenous HIPK2 in RTECs.

### Kinase activity is required for HIPK2-induced downstream pathway activation in RTECs.

We next examined other proinjury and profibrosis pathways potentially mediated by HIPK2 in UUO kidneys. We found that p53 phosphorylation was enhanced in Pax8-HIPK2^WT^ UUO mice but decreased in Pax8-HIPK2^KD^ UUO mice (Supplemental Figure 3, A and B). Additionally, we analyzed marker levels for cell injury (*Kim-1*), cellular proliferation (*Ki-67*), and *Foxm1,* a recently described marker for RTEC repair after injury (15) by quantitative PCR (qPCR). All of these markers were further elevated in the kidney cortices of Pax8-HIPK2^WT^ UUO mice in comparison with Pax8-HIPK2^+/+^ UUO mice (Supplemental Figure 3C). Conversely, in comparison with Pax8-HIPK2^+/+^ UUO mice, their expression was suppressed in Pax8-HIPK2^KD^ UUO mice (Supplemental Figure 3D). In addition to the p53 and TGF-β pathways, HIPK2 also potentiates canonical Wnt and Notch signaling. Therefore, we further examined the expression of Wnt markers and Notch signaling by qPCR. As shown in Supplemental Figure 3E, the expression of *Twist1* and *Axin*2 (canonical Wnt target genes) and *Hey1* and *Heyl* (Notch target genes) was elevated in UUO kidneys in comparison with sham control, and was further elevated in Pax8-HIPK2^+/+^ UUO kidneys. However, their expression was suppressed in Pax8-HIPK2^KD^ UUO kidneys in comparison with Pax8-HIPK2^+/+^ UUO kidneys (Supplemental Figure 3F). Together with the preceding data, these results confirm the key role of HIPK2 as a central regulator of pathways that promote renal fibrogenesis and its function in RTECs as an essential component in driving the fibrosis progression.

### HIPK2 kinase activity and HIPK2-Smad3 interaction are both required for renal fibrosis progression.

**Figure 6:**
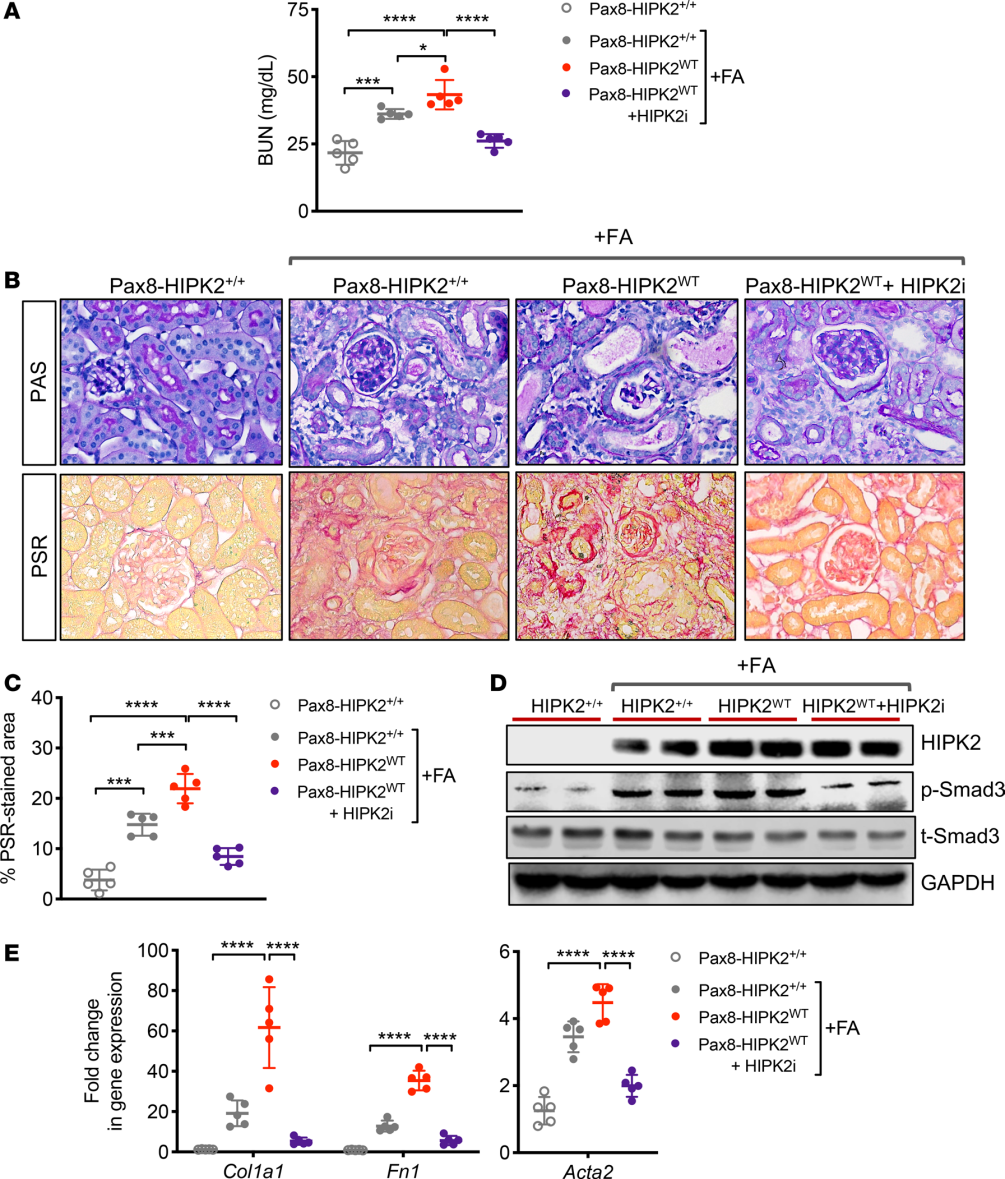
HIPK2 inhibitor (BT173) confers renoprotection in Pax8-HIPK2^WT^ mice folic acid nephropathy. Folic acid (FA) or vehicle control was injected in 8-week-old Pax8-rtTA TRE-hHIPK2 WT mice fed with control chow (Pax8-HIPK2^+/+^) or doxycycline-supplemented chow (Pax8-HIPK2^WT^). A group of FA-injected Pax8-HIPK2^WT^ mice was treated with BT173 (HIPK2i) each day after the FA injection. All mice were euthanized at 28 days after injection. (**A**) Blood urea nitrogen (BUN) levels at day 28 after injection. (**B**) Images of periodic acid-Schiff (PAS)- and picrosirius red (PSR)-stained mouse kidneys with or without FA injection. Scale bar: 20 µm. (**C**) Percentage of PSR-stained area per field (*n* = 30 randomly selected fields per group). (**D**) Representative Western blot images of Pax8-HIPK2^+/+^ and Pax8-HIPK2^WT^ mouse kidney cortices. (**E**) qPCR analysis of fibrosis markers. Data are presented as mean ± SD. *P < 0.05, ***P < 0.001, and ****P < 0.0001 when compared between indicated groups by 1-way ANOVA with Tukey’s multiple comparison test.

We previously showed that the allosteric inhibition of HIPK2 and Smad3 interaction by small-molecule inhibitor BT173 effectively reduces the renal fibrosis in UUO and Tg26 mouse models (13). The preceding results in Pax8-HIPK2^KD^ UUO mice indicated that the HIPK2 kinase activity is also required for fibrogenesis. Therefore, to further assess the mechanism of HIPK2 in mediating the response of RTEC injury, we examined the effect of BT173 in Pax8-HIPK2^WT^ and Pax8-HIPK2^KD^ mice in the setting of folic acid–induced nephropathy (FAN). Eight-week-old Pax8-HIPK2^WT^ and Pax8-HIPK2^+/+^ control mice were injected with folic acid (FA) (250 mg/kg) or vehicle control, and their kidneys were examined 4 weeks after injection. In one set of Pax8-HIPK2^WT^ mice, BT173 was orally administered (20 mg/kg) daily for 4 weeks after FA injection (13). Consistent with the results in UUO and Tg26 mice, Pax8-HIPK2^WT^ mice with FAN showed worsened renal function and more severe renal fibrosis in comparison with Pax8-HIPK2^+/+^ mice with FAN (Figure 6, A–D). However, the administration of BT173 (HIPK2i) markedly improved renal function and attenuation of renal fibrosis in Pax8-HIPK2^WT^ mice with FAN (Figure 6, A–D). Similarly, Smad3 phosphorylation was increased in the Pax8-HIPK2^WT^ mice with FAN as compared with control Pax8-HIPK2^+/+^ mice with FAN, and this was inhibited by BT173 treatment (Figure 6D). qPCR analysis confirmed elevated levels of extracellular matrix genes in Pax8-HIPK2^WT^ mice with FAN compared with Pax8-HIPK2^+/+^ mice with FAN, but reduced levels of the genes in Pax8-HIPK2^WT^ mice with BT173 treatment (Figure 6E).

**Figure 7:**
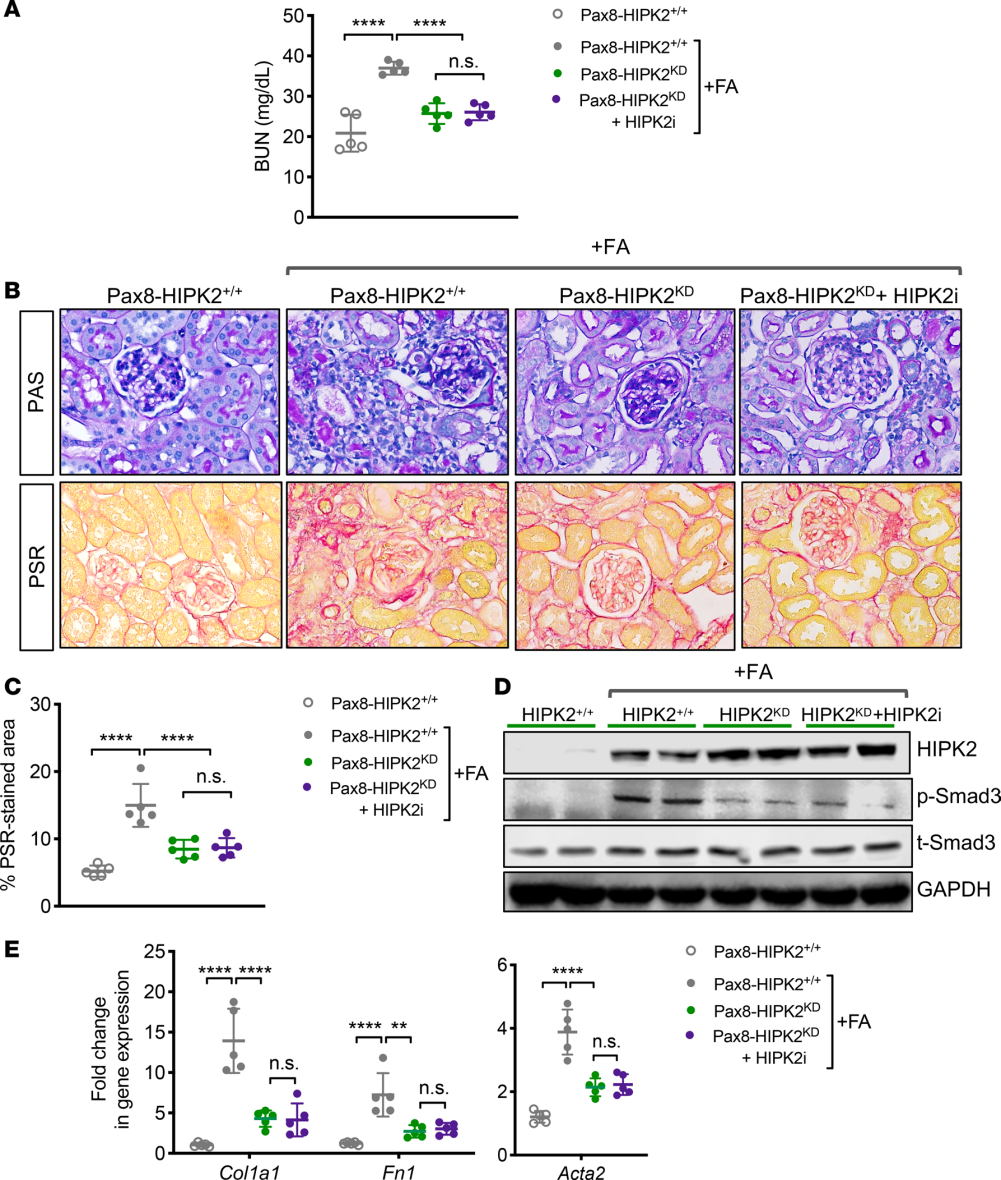
HIPK2 inhibitor (BT173) does not confer increased renoprotection in Pax8-HIPK2^KD^ mice with folic acid nephropathy. Folic acid (FA) or vehicle control was injected in 8-week-old Pax8-rtTA TRE-hHIPK2 KD mice fed with control chow (Pax8-HIPK2^+/+^) or doxycycline-supplemented chow (Pax8-HIPK2^KD^). A group of FA-injected Pax8-HIPK2^KD^ mice was treated with BT173 (HIPK2i) each day after the FA injection. All mice were euthanized at 28 days after injection. (**A**) Blood urea nitrogen (BUN) levels at day 28 after injection. (**B**) Images of periodic acid-Schiff (PAS)- and picrosirius red (PSR)-stained mouse kidneys with or without FA injection. Scale bar: 20 µm. (**C**) Percentage of PSR-stained area per field (*n* = 30 randomly selected fields per group). (**D**) Representative Western blot images of Pax8-HIPK2^+/+^ and Pax8-HIPK2^WT^ mouse kidney cortices. (**E**) qPCR analysis of fibrosis markers. Data are presented as mean ± SD. **P < 0.01 and ****P < 0.0001 when compared between indicated groups by 1-way ANOVA with Tukey’s multiple comparison test.

We next examined the effects of FA injury in Pax8-HIPK2^KD^ mice. Eight-week-old Pax8-HIPK2^KD^ and Pax8-HIPK2^+/+^ mice were injected with FA or vehicle control as previously described and examined after 4 weeks after injection. One set of Pax8-HIPK2^KD^ mice injected with FA received the BT173 treatment for 4 weeks. Notably, the increase in BUN with FAN was not observed in the Pax8-HIPK2^KD^ mice irrespective of BT173 treatment (Figure 7A). The renal fibrosis was increased in control Pax8-HIPK2^+/+^ mice with FAN, but not in Pax8-HIPK2^KD^ mice with FAN with or without treatment of BT173 (Figure 7, B and C). Smad3 phosphorylation and extracellular matrix gene expression followed a similar pattern of changes among these groups of mice (Figure 7, D and E). These data indicate that either the overexpression of HIPK2 kinase-dead mutant act as a dominant-negative in RTECs or the inhibition of HIPK2-Smad3 interaction by BT173 effectively mitigates the renal fibrogenesis in the setting of FAN. No additive effects were observed in Pax8-HIPK2^KD^ mice treated with BT173, indicating that both kinase activity of HIPK2 and interaction of HIPK2 with Smad3 are both required for HIPK2 to promote renal fibrosis.

## Discussion

In the previous study, we used a systems approach to identify HIPK2 as a key driver of kidney fibrosis (12). We also showed that HIPK2 activates multiple profibrosis and proinflammatory pathways in RTECs in vitro. HIPK2 also activates the p53 pathway to induce more RTEC apoptosis. Global KO of HIPK2 attenuates kidney injury and fibrosis in several animal models of kidney disease (12). These studies strongly support the role of HIPK2 in kidney fibrosis. Since our initial publication, several recent studies further support the role of HIPK2 in RTEC injury and renal fibrosis. It has been shown that HIPK2 mediates NOX4 expression in diabetic kidneys (16) and that phosphate niclosamide mitigates renal fibrosis by inhibiting HIPK2 expression in the tubulointerstitial compartment (17). HIPK2 is also involved in acute kidney injury to CKD transition (18).

RTEC injury is considered a critical event that leads to kidney fibrosis. Our previous in vitro studies demonstrated that HIPK2 induces apoptosis and activates profibrosis pathways in RTECs (12). However, whether HIPK2-mediated RTEC injury led to kidney fibrosis in vivo was unclear. Here, we provide strong evidence to support the critical role of HIPK2 in RTECs in the progression of kidney fibrosis in 3 experimental models of renal fibrosis (UUO, Tg26, and FAN). Our data suggest that high HIPK2 expression in human RTECs, as demonstrated in our previous in vitro study (12), likely contributes to the progression of kidney fibrosis. Therefore, pharmacological targeting of HIPK2 could be an attractive strategy to treat kidney fibrosis in patients with CKD.

Notably, without inducing a secondary injury (such as UUO, HIV-infection, or FA injection), the Pax8-HIPK2^WT^ mice did not display any major renal phenotype when examined up to the age of 6 months. This is in contrast with the observation in the transgenic mice with constitutively active TGF-β receptor type 1 kinase that develop prominent tubular cell injury (19), likely as a result of high and continuous activation of the TGF-β pathway, unlike the normal physiological activation of the TGF-β pathway. Our previous data suggest that HIPK2 alone causes only mild TGF-β activation, but that it synergizes with TGF-β signaling to enhance Smad3 activation (20), suggesting that HIPK2 acts as a transcriptional activator of Smad3. Therefore, enhanced tubular injury and renal fibrosis develop in Pax8-HIPK2^WT^ mice in response to a high endogenous TGF-β activity, as seen in the UUO or FAN kidneys (a second hit).

HIPK2 is an evolutionarily conserved serine/threonine nuclear kinase comprised of several domains, including a 330 amino acid Ser/Thr kinase domain, in which K228 is the key catalytic site. Several studies have investigated the importance of the kinase domain in HIPK2. For instance, Huang and colleagues showed that the phosphorylation of S359/T360 of HIPK2 in its kinase domain is an essential link that connects the IRE1a pathway to JNK activation and neuronal cell death under the ER stress (21), and the JUN pathway is critical for kidney cell injury and fibrosis (22). HIPK2 kinase activity is also required for Hippo pathway regulation (23), the dysregulation of which is implicated in kidney injury and fibrosis (24–27). In our previous study, we demonstrated that overexpression of a kinase-dead mutant of HIPK2 in cultured RTECs substantially reduced the HIV-induced apoptosis and diminished the expression of profibrotic markers in vitro (12). Our current study now provides the in vivo evidence that kinase activity of HIPK2 is required to promote renal fibrogenesis for full activation of Smad3, p53, Notch, and Wnt pathways in the diseased kidney.

However, a major limitation of HIPK2 as a drug target is its inhibition of the p53 pathway (28–30). The regulation of the p53 pathway was shown to depend on the HIPK2 kinase activity and the interaction of p53 with the sumoylation site on HIPK2 (28–31). Although the inhibition of p53 in RTECs may promote and maintain cell survival, its inhibition may increase the possibility of tumorigenesis. To avoid its effects on p53, we developed a small molecule, BT173, which specifically inhibits the HIPK2-Smad3 interaction allosterically, without affecting the HIPK2 kinase activity (13). We showed in cultured renal epithelial cells that both HIPK2 activity and its interaction with Smad3 are critical for TGF-β–mediated gene expression in vitro, and that BT173 abolished Smad3 activation and attenuated kidney fibrosis without affecting p53 activation in UUO and Tg26 mice (13). Our data indicate that overexpression of HIPK2^KD^ and treatment of BT173 reduced the renal fibrosis to a similar extent in mice with FAN, and that administration of BT173 in Pax8-HIPK2^KD^ mice did not provide additional benefit, suggesting that both HIPK2 activity and its interaction with Smad3 are required for its profibrosis effect.

In summary, these data collectively suggest inhibitors of HIPK2, either inhibiting the kinase activity or its interaction with downstream effectors such as Smad3, would be therapeutically effective against renal fibrosis in CKD. However, the inhibition of HIPK2 and Smad3 interaction would confer specific anti-fibrosis effect without the alteration of necessary signaling pathways such as p53. Future studies are required to further map the domains of HIPK2 responsible for its activation of proapoptosis and profibrosis pathways. These mechanistic studies will allow for the development of better HIPK2 inhibitors as therapy for kidney fibrosis.

## Methods

### Generation of RTEC-specific HIPK2-null mice.

We obtained a strain of “KO first, conditional ready” HIPK2 mice from the European Mutant Mouse Archive (05113, C57BL/6NTac-Hipk2^tm2a[EUCOMM]Hmgu/Cnrm^). In this strain, the targeting cassette of the KO-first allele is flanked by flippase recognition target recombination sites and inserted into the intron of *Hipk2* gene, which contains an *IRES:LacZ* trapping cassette and a *fl* promoter-driven *neo* selection cassette. In addition to the disruption cassette, this allele also has 2 loxP sites that flank the critical exons, 3 and 4, which encode the first ATG and entire HIPK2 kinase domain (from amino acid 192 to 520). Heterozygous mice with *Hipk2*-KO–first allele were first bred with Flp transgenic mice (JAX stock no. 011065) to generate mice with *Hipk2* *fl* conditional allele. These mice were then crossed with transgenic mice carrying Pax8-driven rtTA and TRE-Cre transgenes.

### Generation of RTEC-specific HIPK2 WT transgenic mice.

Human HIPK2 cDNA corresponding to NM_022740.4 was purchased from GeneCopoeia Inc. A fusion C-terminal 6xHis-tagged HIPK2 was generated by PCR and subcloned into the pGEM-T Easy plasmid (Promega Life Science). The HIPK2 fusion construct was then further modified to express an enhanced GFP (EGFP) bicistronically, named pTRE-Tight-hHIPK2-HIS_6_-IRES-EGFP. The sequence and orientation of the HIPK2-HIS_6_ insert were confirmed by restriction endonuclease and DNA sequencing. The dox-inducible expression of HIPK2-HIS_6_ was confirmed in the Tet-ON U2OS cell line. The 6.5-kb pvu1 DNA fragment of pTRE-Tight-hHIPK2 WT-HIS_6_-IRES-EGFP was used for microinjection to generate the TRE-hHIPK2 WT transgenic mice in the FVB/N background. Similarly, the DNA fragment of pTRE-hHIPK2 KD-IRES-EGFP was used for microinjection to generate the TRE-hHIPK2 KD transgenic mice in the FVB/N background. These were crossed with Pax8-rtTA to generate tubular epithelial cell-specific overexpression of HIPK2-WT or HIPK2-KD. HIPK2 overexpression was validated by Western blot analysis of kidney cortices from these mice after 3 weeks of dox-supplemented chow feeding.

### Generation of RTEC-specific HIPK2 kinase-dead transgenic mice.

The HIPK2 WT DNA fragment was amplified by PCR using 2 sets of primers (HHIPK2K228): 5′-CAATGAGATCGTAGCCATCCGTA-3′ (forward) and 5′-ATCGTAGCCATCCGTATCCTGAAGAACC-3′ (reverse) and purified from 2% agarose gels. Mutation screening for HIPK2 was carried out by prescreening with denaturing high performance liquid chromatography analysis (WAVE DNA Fragment Analysis System, Transgenomic) and sequenced using a Big Dye Terminator Sequencing Kit and ABI Prism 3100 Genetic Analyzer.

### UUO model.

To create the UUO model, the left ureter of mice was exposed through a midline abdominal incision and ligated using 4-0 silk. Contralateral sham-operated kidneys (ureters exposed, but not ligated) were used as controls. All surgeries were performed under general anesthesia with isoflurane.

### FAN model.

Pax8-HIPK2^WT^, Pax8-HIPK2^KD^, and control Pax8-HIPK2^+/+^ mice were injected intraperitoneally with 250 mg/kg FA (MilliporeSigma) or equivalent volumes of vehicle control 3 weeks after with or without dox feeding. All mice were euthanized 4 weeks after FA or vehicle control injection. In a subset of mice, 20 mg/kg BT173 were administered orally as described previously (13), starting the day after FA injection for 4 weeks.

### Western blot.

Protein lysate preparation and Western blot analysis were performed as previously described. Briefly, tissues were lysed with buffer containing 1% Nonidet P-40 and a protease and phosphatase inhibitor cocktail. The specific antibodies described below were used for immunoblot analysis. Antibody against HIPK2 was purchased from Abcam (ab28507). Antibodies for p-p53, phospho-Smad3 (9520), total Smad3 (9513), and GAPDH (2118) were purchased from Cell Signaling Technology. The density for each tested protein was normalized against GAPDH.

See complete unedited blots in the supplemental material.

### Urine albumin and creatinine measurements.

Urine protein was measured using a commercial assay ELISA kit (Bethyl Laboratory). Urine creatinine levels were quantified using a QuantiChrom Creatinine Assay Kit (DICT-500, Bioassay System). Urine albumin excretion was expressed as an albumin-to-creatinine ratio.

### qPCR.

Samples were stored in RNAlater (QIAGEN) solution at –80°C until processing. Total RNA was extracted from kidney cortices using the RNeasy Kit (QIAGEN). Superscript III First-Strand Synthesis SuperMix (Invitrogen) was used for reverse transcription of 1 μg total RNA. Quantitative real-time PCR was performed using SYBR Green Master Mix (Applied Biosystems) and the 7500 Real-Time PCR System (Applied Biosystems). Intron-spanning primer sets selectively targeting mRNA and not the genomic DNA sequence were designed using Primer-BLAST (NCBI). Gene expression was normalized to the housekeeping gene GAPDH, and fold changes in expression relative to the control group were calculated using the 2^−ΔΔCT^ method, where C_T_ is the cycle threshold.

### PSR and Masson’s trichrome staining.

Renal fibrosis was evaluated histologically by Sirius red staining (ab 150681; Abcam) and Masson’s trichrome staining (87019; Thermo Scientific) on paraffin slides of mouse kidney tissues. Six fields were randomly selected in the renal cortex area per mouse kidney. PSR -stained slides were analyzed using methods previously described using Image J.

### Statistics.

Data are presented as mean ± SD. A 1- or 2-way ANOVA with Tukey’s multiple comparison test was used to analyze means between groups. GraphPad Prism 7 software was used for statistical analyses. *P* values less than 0.05 were considered statistically significant.

### Study approval.

All animal studies were performed according to the protocols approved by the Institutional Animal Care and Use Committee at the Icahn School of Medicine at Mount Sinai (IACUC-LA09-00377).

## Author contributions

WX, KL, and JCH designed the experiments. WX, JE, LB, AW, ZL, and RL performed the experiments. YF, YJ, DB, ZL, YLZ, RL, KL, and JCH analyzed the data. WX, KL, and JCH drafted and revised the manuscript.

## Supplementary Material

Supplemental data

## Version 1 07/23/2020

In-Press Preview

## Version 2 09/03/2020

Electronic publication
