# Supplementary material for: Tubular HIPK2 is a key contributor to renal fibrosis
Source: JCI Insight. 2020 Sep 3;5(17):e136004. doi: 10.1172/jci.insight.136004 (PMC7526443; doi:10.1172/jci.insight.136004)
Supplement: Supplemental data [file jciinsight-5-136004-s176.pdf]

Suppl. Figures

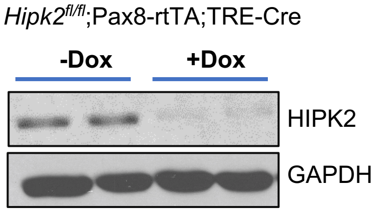

S. Figure 1: **Effective RTEC-specific HIPK2 ablation with doxycycline administration**. 6-week old *Hipk2*$^{fl/fl}$;Pax8-rtTA; TRE-Cre mice were given control (-Dox) or doxycycline-supplemented chow (+Dox, 625mg/kg) for three weeks. Western blot was performed of kidney cortices from two mice with or without Dox supplementation.

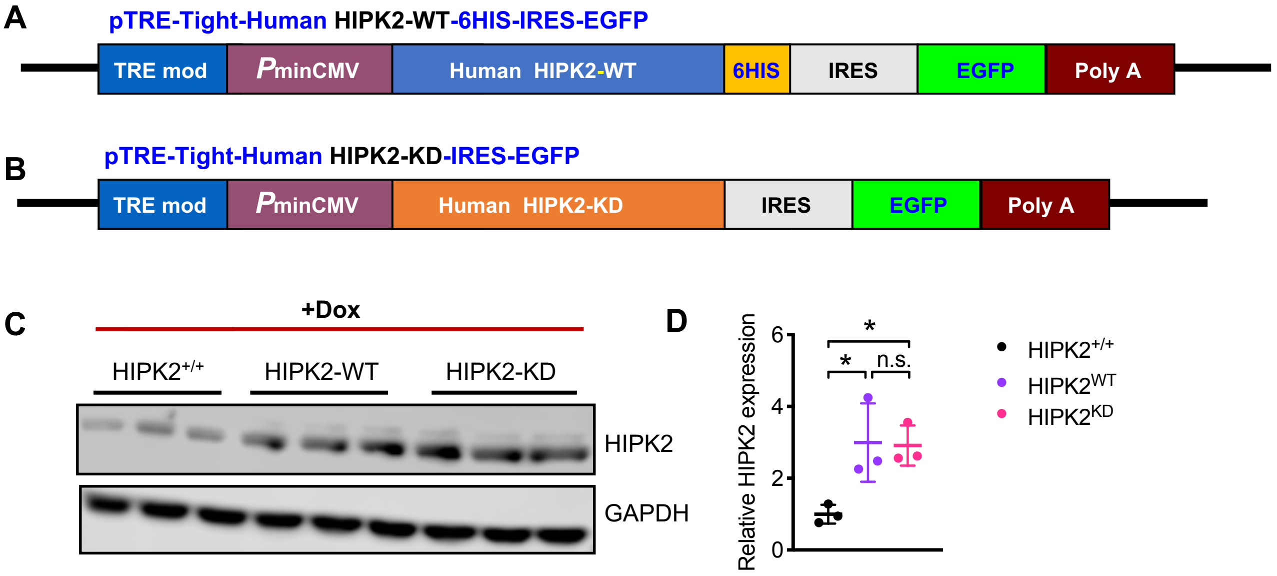

S. Figure 2: **Effective RTEC-specific HIPK2 overexpression with doxycycline administration**. (A-B) Schematics of the tetracycline-inducible vector expressing (A) His_6-tagged human HIPK2 WT and (B) HIPK2 kinase dead (KD) proteins. (C) 6-week old HIPK2 wildtype control (HIPK2$^{+/+}$), Pax8-rtTA;TRE-HIPK2-WT (HIPK2-WT), and Pax8-rtTA;TRE-HIPK2-KD (HIPK2-KD) mice were given doxycycline-supplemented chow (+Dox, 625mg/kg) for three weeks. Western blot was performed of kidney cortices of mice for total HIPK2 protein expression. (D) Densitometric analysis of HIPK2 protein expression in three groups of mice is shown as average fold change relative to HIPK2$^{+/+}$ control (n=3 mice per group, data are expressed as mean±SD, *$P<0.05$ 1-way ANOVA with Tukey's multiple comparison test.

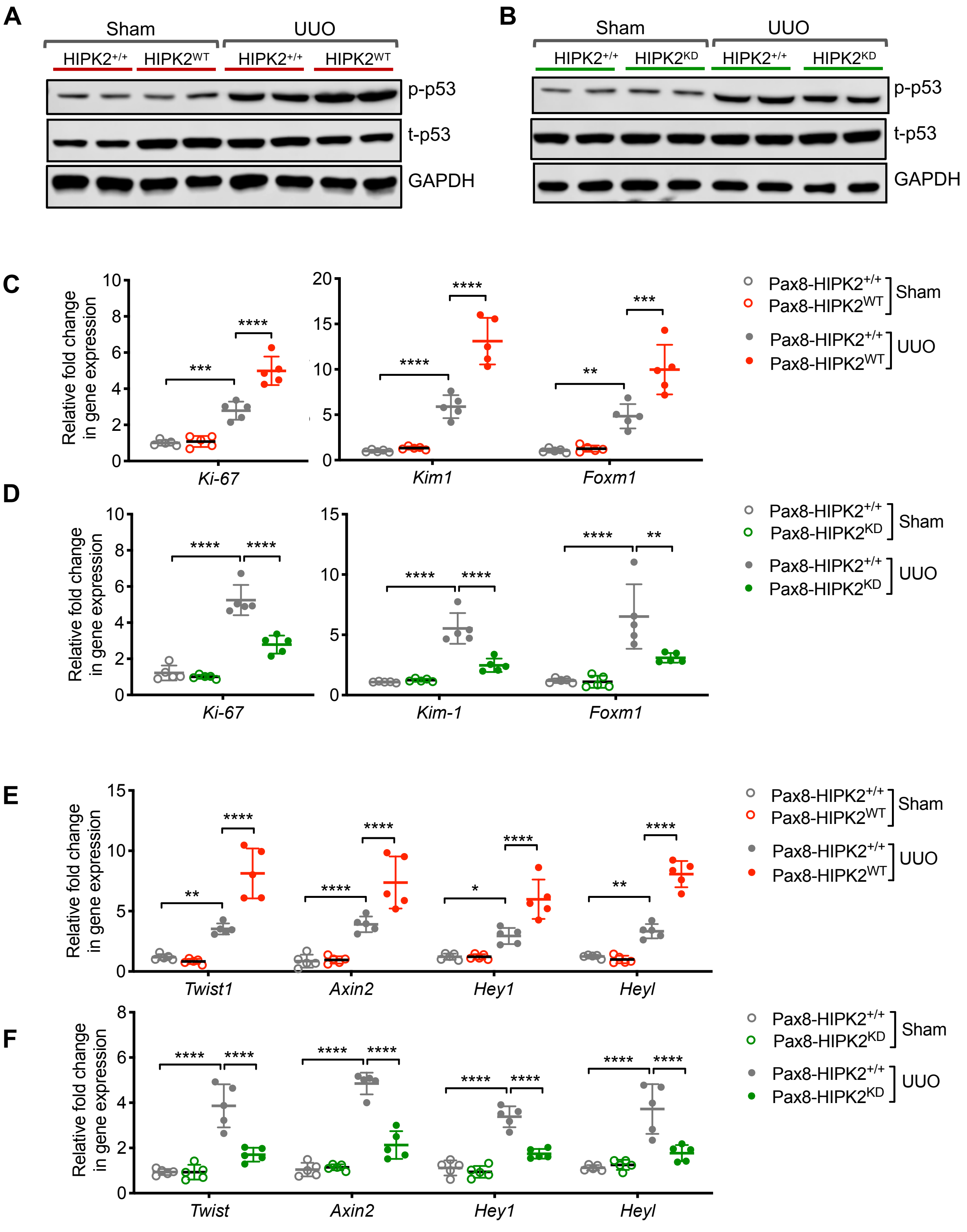

S. Figure 3: **HIPK2 potentiates p53, canonical Wnt, and Notch signaling in UUO kidneys.** (A-B) Western blot analysis of phosphorylated p53 (p-53) and total p53 (t-p53) in control of UUO kidneys of Pax8-HIPK2^{WT} mice (A) or Pax8-HIPK2^{KD} mice (B) in comparison to control Pax8-HIPK2$^{+/+}$ mice (without doxycycline-supplemented chow). Western blot was performed of kidney cortices from two mice from each group. (C-D) Quantitative PCR analysis of *Ki-67*, *Kim-1*, and *Foxm1* in control of UUO kidneys of Pax8-HIPK2^{WT} mice (C) or Pax8-HIPK2^{KD} mice (D) in comparison to control Pax8-HIPK2$^{+/+}$ mice. (E-F) Quantitative PCR analysis of canonical Wnt target genes (*Twist* and *Axin2*) and Notch target genes (*Hey1* and *Heyl*) in control of UUO kidneys of Pax8-HIPK2^{WT} mice (E) or Pax8-HIPK2^{KD} mice (F) in comparison to control Pax8-HIPK2$^{+/+}$ mice. All data are expressed as mean±SD, *P<0.05, **P<0.01, ***P<0.001, and ****P<0.0001 when compared between indicated groups by 1-way ANOVA with Tukey's multiple comparison test.
